# Surface defect detection of hot rolled steel based on multi-scale feature fusion and attention mechanism residual block

**DOI:** 10.1038/s41598-024-57990-3

**Published:** 2024-04-01

**Authors:** Hongkai Zhang, Suqiang Li, Qiqi Miao, Ruidi Fang, Song Xue, Qianchuan Hu, Jie Hu, Sixian Chan

**Affiliations:** 1 School of Electronic and Information Engineering, Anhui Jianzhu University, Hefei, 230601 China; 2 Key Laboratory for Comprehensive Energy Saving of Cold Regions Architecture of Ministry of Education, Jilin Jianzhu University, Changchun, 130119 China; 3 Department of Information Engineering and Art Design, Anhui Zhong-Ao Institute of Technology, Hefei, 230041 China; 4 Key Laboratory of Intelligent Informatics for Safety and Emergency of Zhejiang Province, Wenzhou University, Wenzhou, 325035 China; 5 College of Computer Science and Technology, Zhejiang University of Technology, Hangzhou, 310023 China

**Keywords:** Mechanical engineering, Computer science, Information technology

## Abstract

To improve the precision of defect categorization and localization in images, this paper proposes an approach for detecting surface defects in hot-rolled steel strips. The approach uses an improved YOLOv5 network model to overcome the issues of inadequate feature extraction capacity and suboptimal feature integration when identifying surface defects on steel strips. The proposed method achieves higher detection accuracy and localization precision, making it more competitive and applicable in real production. Firstly, the multi-scale feature fusion (MSF) strategy is utilized to fuse shallow and deep features effectively and enrich detailed information relevant to target defects. Secondly, the CSPLayer Res2Attention block (CRA block) residual module is introduced to reduce the loss of defect information during hierarchical transmission, thereby enhancing the extraction of fine-grained features and improving the perception of details and global features. Finally, the experimental results indicate that the mAP on the NEU-DET and GC10-DET datasets approaches 78.5% and 67.3%, respectively, which is 4.9% and 2.1% higher than that of the baseline. Meanwhile, it has higher precision and more precise localization capabilities than other methods. Furthermore, it also achieves 59.2% mAP on the APDDD dataset, indicating its potential for growth in further domains.

## Introduction

In the industrial production of hot-rolled steel strips^1–7^, defect detection is a crucial task in the manufacturing domain. Its primary objective is to use automation and computer vision techniques to detect and identify defects, flaws, or anomalies in the manufacturing process. This plays a pivotal role in ensuring product quality, enhancing production efficiency, and reducing costs. Industrial defect detection has undergone four main stages: manual inspection, image processing, machine learning, and deep learning. In the deep learning stage, the rapid advancements in Convolutional Neural Networks (CNNs)^8–10^ have led to great performance improvements in industrial defect detection. CNNs can realize efficient and accurate defect detection through automated learning and feature extraction from images^11–15^.

**Figure 1:**
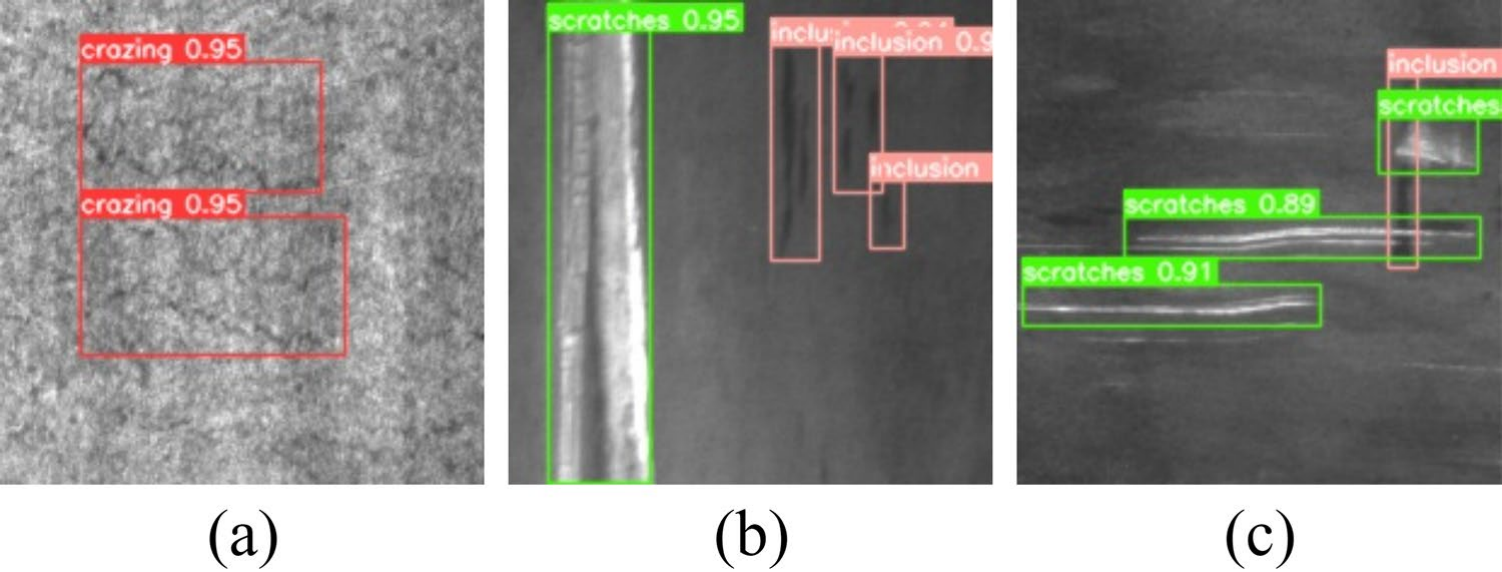
Complex defects on the hot rolled steel surface: (**a**) multiple defects, (**b**) multiple classes of defects, (**c**) overlapping defects.

Defect detection is a specialized branch in computer vision tasks, and it is a process through which a computer identifies the presence and location of defects in images and annotates them with defect category labels, confidence scores, and bounding boxes delineating the defect’s position. For the task of detecting surface defects on steel strips, the challenge extends beyond the identification of single-class defects within individual images, as shown in Fig. 1a. It involves the detection of multiple defect classes within the same image, as shown in Fig. 1b, and the identification of overlapping instances of multiple defect types within a single image, as shown in Fig. 1c. At present, defect detection algorithms based on deep learning convolutional neural networks face a series of difficulties and challenges, including a lack of samples in steel strip surface defect datasets, an uneven distribution of samples, overly simplistic classification tasks, and inaccuracy in defect position annotations^16,17^.

This paper mainly alleviates the following challenges. In the process of feature extraction using CNNs for accurate defect identification and precise localization, there are significant challenges. Some defect information in images may be lost as they traverse through the convolutional layers of CNNs, potentially causing reduced detection accuracy and inaccurate target localization^18,19^. Deep learning-based methods usually address this issue by fusing feature maps from multiple different levels^7,20–23^. However, this multiscale feature fusion approach also has some problems^24–28^. Shallow feature maps provide higher resolution and detailed information but have weaker discriminative power, while deep feature maps provide higher-level semantic information but lack sensitivity to details^29^. Therefore, in the feature fusion process, there are issues related to information loss and inaccurate localization. This paper achieves comprehensive feature utilization by merging feature maps from different levels and exploiting their advantages in terms of resolution and semantic information. The combination of shallow feature maps with deep feature maps provides both more detailed information and higher-level semantic representation, leading to feature maps with both high resolution and strong semantic representational capabilities. This helps to better capture both detail and semantic information within the images, thereby enhancing model performance. Though most existing residual blocks have achieved success in deep learning^30–33^, optimization is needed for practical industrial defect detection applications, especially for small-scale object detection and fine-grained feature extraction. Introducing residual blocks can address some of the information loss issues, but an emphasis on detailed information is still lacking, with insufficient fine-grained feature extraction capabilities. This paper introduces the CRA block, which incorporates an attention mechanism to assist the network model in focusing on critical regions and features within the images when handling defect information. It adaptively adjusts the weights of various branches, making them more sensitive to defect information, thereby improving the model’s perception and feature extraction capabilities for defects, ultimately improving defect detection accuracy and effectiveness. In industrial production processes, due to strict quality requirements, collecting representative defect datasets is extremely challenging. The quantity, categories, and complexity of defects play a crucial role in evaluating a model’s defect detection capabilities. Since the occurrence frequency of specific defects is low, it is difficult to obtain a sufficient variety of defect samples, which may result in a model that lacks generalization ability and cannot accurately detect and classify various defects in real-world scenarios. Models with robust detection capabilities and strong generalization performance are required to overcome the great challenge of accurately detecting and localizing complex defects. This paper performs a comprehensive and reliable performance evaluation of the improved model using two defect datasets collected from real-world scenarios, and the study results provide insights into the model’s performance in practical applications.

The primary contributions of this paper are summarized as follows:

- The MSF strategy is proposed to enhance the faulty features’ representation capacity. It efficiently increases image resolution while acquiring more robust semantic information by combining the benefits of feature maps of various scales.
- The CRA block is introduced, which combines an attention mechanism with residual and skip connections to better capture multi-scale feature information and improve feature fusion, leading to increased awareness of general aspects and finer details.
- The model’s performance is validated using the NEU-DET, GC10-DET and APDDD datasets, and the results demonstrate the great capabilities of the improved YOLOv5s model.

The rest of this paper is divided into the following sections. The “Related work” section introduces related research on attention-based residual blocks, multi-scale feature fusion, and deep learning applied to strip defect detection. The proposed fusion approach and residual blocks are described in detail in the “Methodology” section. The experimental data, results, and visual analysis of these results are provided in the “Experiments and analysis” section. The significance of enhancing the precision of detecting tiny flaws and the constraints of current studies are elucidated in the “Discussion” section. Finally, the approach proposed in this study is further discussed and evaluated in the “Conclusion” section.

## Related work

### Deep learning application for steel strip defect detection

Deep learning, particularly CNNs, has been extensively used in the field of steel strip defect detection, gradually replacing traditional machine vision-based methods. These algorithms have gained widespread application in various industrial domains, including the detection of metal surface defects and PCB board defects. Deep learning models based on CNNs for defect detection can be categorized into two-stage and one-stage algorithms according to their structural differences. Two-stage detection models include R-CNN^34^, SPPNet^35^, Fast R-CNN^36^, Faster R-CNN^37^, and Mask R-CNN^38^, which divide the defect detection task into two phases: generating candidate regions and classifying and locating defects. These models usually provide higher detection accuracy. For instance, Liu et al.^39^ improved the Faster R-CNN network by utilizing multiscale feature fusion and guided anchor RPN to realize adaptive anchor adjustment, addressing issues like complex texture interference and small defect sizes on uneven solar cell surfaces, significantly increasing defect detection accuracy. Xu et al.^40^ proposed a novel tunnel defect detection method based on Mask R-CNN to overcome the challenges of time-consuming and error-prone manual inspections. One-stage models include the YOLO series^41–46^ and SSD^47^, which regard object detection as a regression problem. These models have fast inference speeds and support real-time object detection but may have slightly lower detection accuracy compared to two-stage models. Researchers have improved these models to increase defect detection accuracy by introducing techniques such as multiscale feature fusion and adaptive anchor adjustment. For instance, Liu et al.^48^ enhanced YOLOX with a parallel fusion network structure and a self-adjusting label assignment algorithm and applied the model to address the differentiated semantic hierarchy of defects in images and the dynamic changes during model training, thereby significantly improving defect detection accuracy. Cheng et al.^21^ proposed a RetinaNet with differential channel attention and adaptive spatial feature fusion to enhance accuracy for specific defect categories and precise defect localization in steel surface defect detection. To sum up, one-stage models are more suitable for applications requiring high speed and real-time processing, while two-stage models are better suited for applications demanding higher detection accuracy and precision. Therefore, it is necessary to conduct further research to enhance steel strip defect detection accuracy while maintaining a high detection speed.

### Fusion of multiple-scale features

The feature pyramid is a classic approach in object detection, and it can exploit multiscale feature information. In previous research, many object detection models that directly use image features extracted by the backbone network were proposed. However, to enable the complementary and fusion of features at different scales, thereby improving the performance of object detection and segmentation, the concept of a feature pyramid was introduced. Methods such as FPN^24^, PANet^25^, two-way FPN^26^, etc. enhance model performance by fusing features of different scales through top-down or bottom-up pathways. EfficientDet^49^ introduces a repeatable BiFPN for iterative feature fusion, further enhancing detection performance. NAS-FPN^27^ and Auto-FPN^28^ employ optimization techniques such as reinforcement learning or evolutionary algorithms to search for the optimal feature pyramid network structure to automatically discover and design more efficient feature pyramid networks. These methods have greatly improved the performance of object detection, providing new insights and approaches for feature fusion and network architecture design.

### Blocks with residual based on attention mechanism

In defect detection, residual blocks play a crucial role in improving the depth and performance of models. The concept of residual blocks was introduced by He et al.^50^, enabling networks to model deeper levels and enhancing feature representation capabilities. Skip connections can address the issue of gradient propagation. Attention mechanisms help the model to focus on areas where defects may exist, thereby enhancing detection rates and localization accuracy. In defect detection tasks, the introduction of attention-based residual blocks is highly significant. Firstly, due to the diversity and complexity of defects, deeper models are needed to extract rich feature representations. Residual blocks enable deeper modeling and thus enhance the model’s expressive power. Secondly, residual blocks, through skip connections, can directly transmit information from shallow layers to deep layers, thereby providing a stronger gradient flow path while maintaining the effectiveness of low-level features. Finally, attention mechanisms make the model focus more on areas where defects may exist, leading to enhanced defect detection rates and localization accuracy. Xue et al.^51^ introduced a novel hierarchical residual network with an attention mechanism for spectral-spatial classification of hyperspectral images. The incorporation of attention mechanisms into residual blocks improves the model’s learning and feature representation capabilities, allowing the model to better capture and distinguish defect features. Liu et al.^52^ introduced residual blocks with coordinated attention mechanisms to reduce feature information loss and accelerate the detection progress. Thus, in current defect detection research, attention-based residual blocks are widely used in various deep learning models, making great contributions to improving the accuracy and robustness of defect detection.

## Methodology

In industrial defect detection tasks, both one-stage and two-stage detection models are widely utilized. One-stage models have advantages such as high real-time performance, simplicity, and speed, so they are suitable for industrial applications rerunning real-time response and high efficiency. Meanwhile, two-stage models provide higher detection accuracy and precise localization capabilities, making them suitable for detecting small defects in complex scenes. Based on the requirements of industrial defect detection, YOLOv5s is chosen as the baseline in this study because of its advantages in terms of lightweight design, multi-scale detection, data augmentation, and ease of deployment. In industrial defect detection, YOLOv5s can detect and locate various defects rapidly and accurately, thereby improving production quality and efficiency.

**Figure 2:**
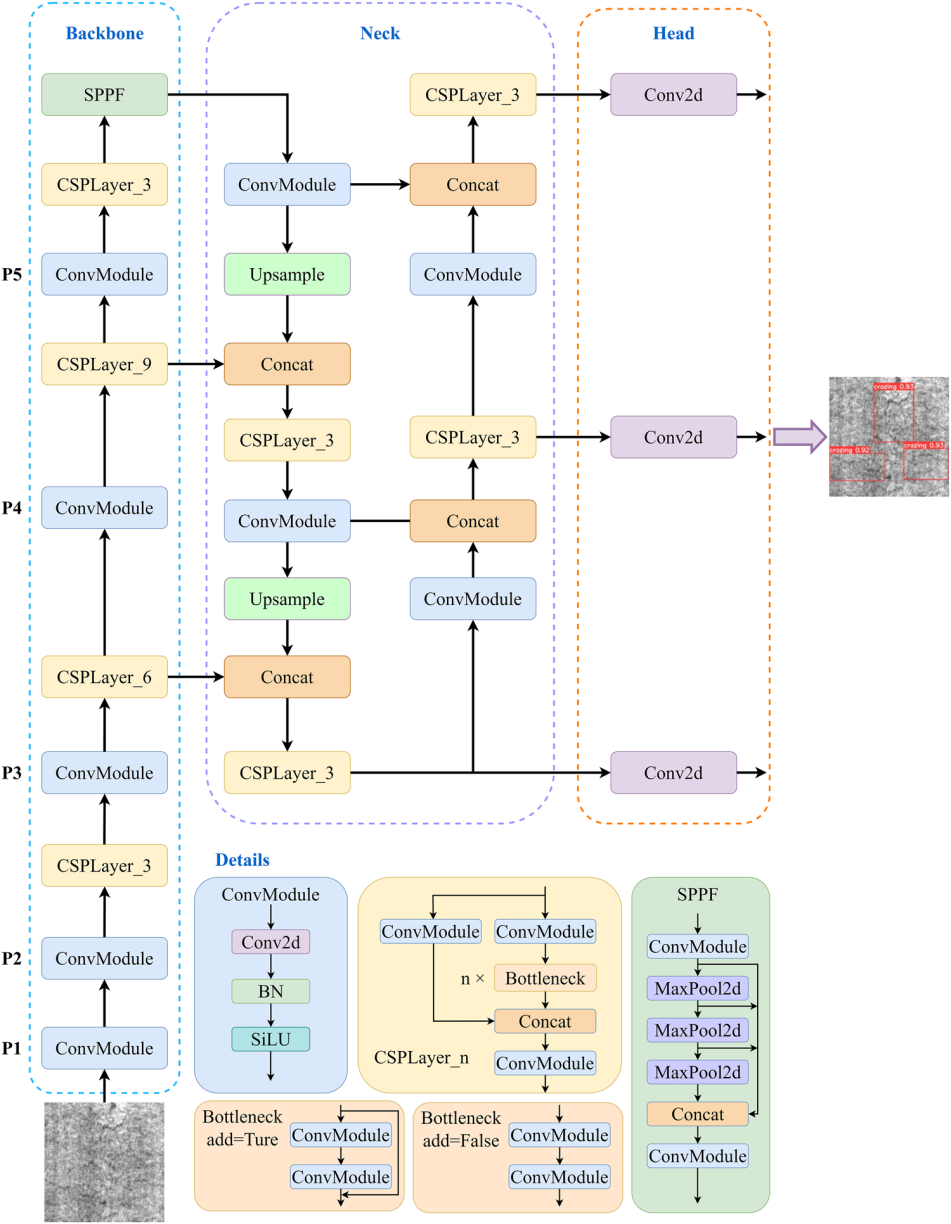
YOLOv5’s overall architecture diagram.

### Baseline network architecture

YOLOv5 is a single-stage object detection algorithm based on anchors, and it is designed to provide a high-performance, high-speed, and high-accuracy framework for object detection. YOLOv5 has many versions, including YOLOv5-n, YOLOv5-s, YOLOv5-m, YOLOv5-l, and YOLOv5-x, where the “n”, “s”, “m”, “l”, and “x” suffixes represent variations in network architecture and parameter quantities. As the depth and width of the network increase, the detection accuracy also improves, but it comes at the cost of reduced speed. YOLOv5s consists of three main components: the Backbone, the Neck, and the YOLO Head, as illustrated in Fig. 2. YOLOv5 utilizes the CSPDarkNet53 and SPPF modules as the backbone network for feature extraction from images. CSPDarknet53 is a combination of Darknet and CSPNet. It divides the input features into two paths and connects them through an intermediate CSP module to achieve better feature representation capability. The SPPF module consists of three sequential MaxPooling layers with a convolutional kernel size of 5$\times$5. This module is utilized to further aggregate and strengthen the features extracted by the backbone network. It can capture contextual information of the target at different receptive fields, thereby providing more expressive feature representations for subsequent object detection tasks. YOLOv5 combines PAN in a manner that facilitates the propagation of semantic information from top to bottom and the transmission of detailed information from bottom to top. This implementation is conducive to multi-scale feature fusion, thereby providing the network with more comprehensive and representative feature information. By utilizing the PAN structure, YOLOv5 can better integrate feature information across various scales, thereby providing a more robust representation of both semantic and fine-grained details. The YOLO Head is a crucial component in YOLOv5, and it is responsible for performing object detection tasks on the features extracted from the backbone network. It achieves this by defining prediction boxes using anchor boxes and utilizing an independent logistic classifier for category prediction. Meanwhile, it refines the bounding box coordinates and dimensions through regression for precise localization. Finally, the final object detection results are obtained by applying thresholding and non-maximum suppression.

### Improvement of YOLOv5s network architecture design

#### The multiscale feature fusion approach

**Figure 3:**
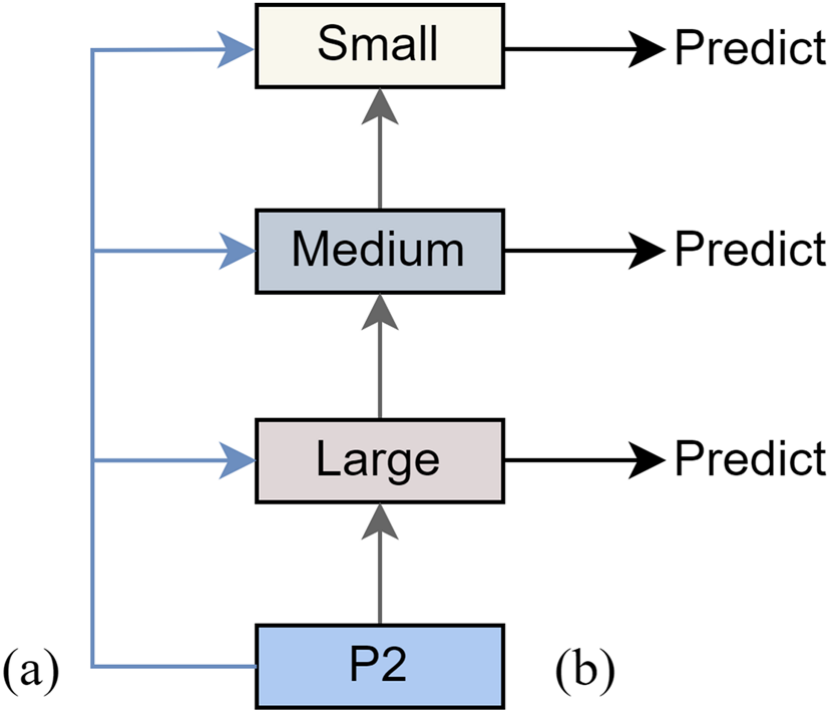
Multiscale feature fusion approach: (**a**) the gray lines represent the fusion of the P2 feature layer with the detection layer separately, (**b**) the blue lines represent the fusion of the P2 feature layer with the detection layer successively.

The feature information used for detecting small and medium-sized objects are intertwined at the lower level (P2) of the FPN. Though different levels of the pyramid contain size-specific object information, current feature fusion methods usually neglect high-resolution shallow layers, resulting in difficulties in detecting small objects. To address this issue, this study proposes a new fusion technique called MSF, as shown in Fig. 3. MSF aims to leverage shallow layers effectively and enhance the detection of small objects. To combine the P2 feature map with semantically enhanced feature layers, MSF provides two fusion techniques, and the combination of them can improve the feature map’s resolution while retaining more robust semantic information. The P2 feature layer and detection layer are combined to reduce information loss, improve multi-scale and small object identification capabilities, increase the accuracy of detecting objects of various sizes, and further improve feature representation. Defects in steel strips are often small-scale and contain specific information, such as “crazing” and “rolled-in scale” in the NEU-DET dataset, “Punching” and “Inclusion” in the GC10-DET dataset. Therefore, it is crucial to fully exploit the P2 layer retrieved by the YOLOv5s backbone network to better capture the specifics and small-scale target information in defect images.

#### CRA block

**Figure 4:**
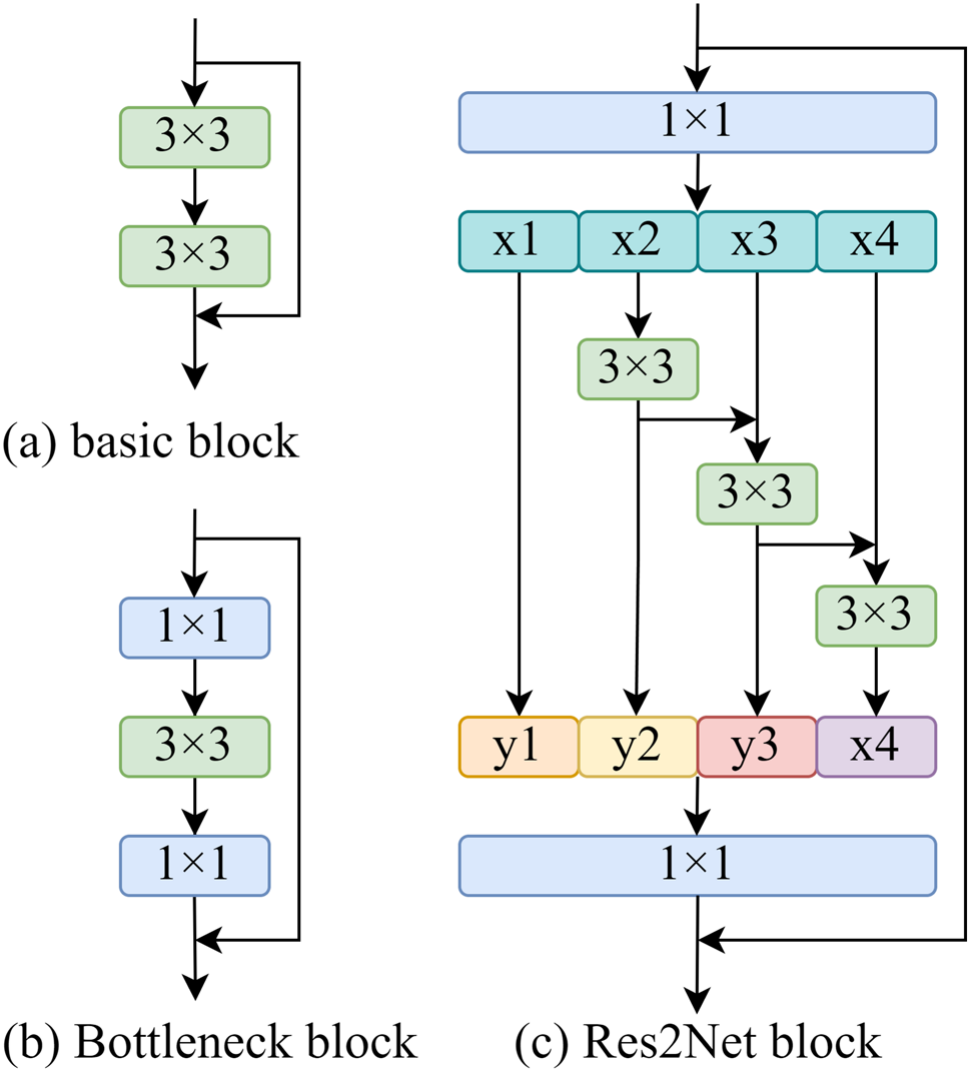
Comparison of different residual blocks.

**Figure 5:**
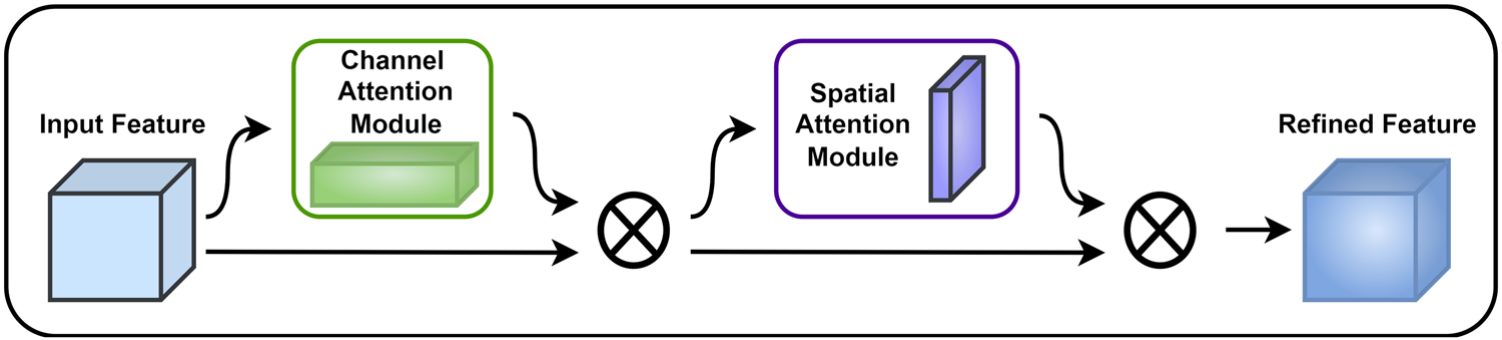
The overall structure of CBAM consists of CBAM modules, including the channel attention module and spatial attention module.

**Figure 6:**
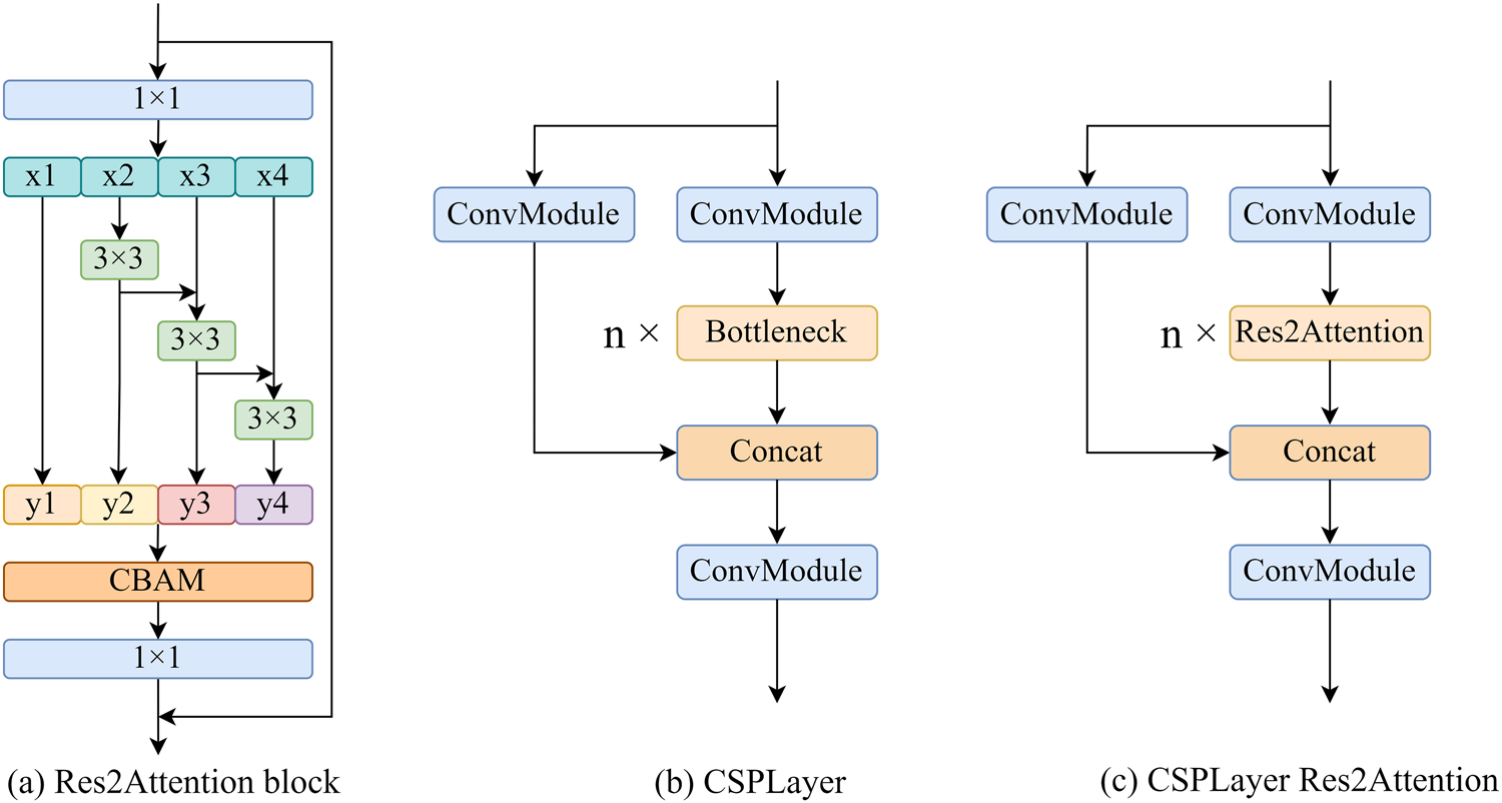
Comparison of three residual blocks: (**a**) Res2Attention block, (**b**) CSPLayer, (**c**) CRA block. The configurable scaling dimension of the Res2Attention block is set to 4.

Residual blocks are a common choice for model architectures in the current work. Figure 4 shows a comparison between three commonly used residual blocks: the Basic block, the Bottleneck block, and the Res2Net block^53^. These blocks are incorporated into various model architectures currently in use. However, these residual block models might be susceptible to noise interference, leading to the loss of fine details throughout the processing and limiting the model’s performance. The Res2Attention block is shown in Fig. 6a, the CSPLayer is shown in Fig. 6b, and both are integrated into the CRA block, as illustrated in Fig. 6c, to mitigate the detrimental effects that noise has on the performance of residual block models. By expanding the receptive field and adding the CBAM attention module, as illustrated in Fig. 5, the CRA block overcomes the limitations of receptive fields and information loss during feature extraction, making the model have higher robustness. Besides, skip connections and residual connections work together to reduce overfitting while improving feature fusion and information interaction capabilities. Additionally, the addition of attention processes and residual connections enhances feature extraction, model stability, and generalization capacity.

The CRA block applies a 1Ã-1 convolution to split the channel dimension of the input feature map into *S* groups, denoted as $x_{1},x_{2},...,x_{S}$. Although the number of channels is decreased to 1/*S* of the original, each feature map subset $x_{i}$ has the same spatial size. Meanwhile, a 3Ã-3 convolution operation is used in each group, except for $x_{1}$, and it is designated as $K_{i} (~)$. With the exception of $x_{1}$ and $x_{2}$, the feature map $x_{i}$ is added with the output of $K_{i-1} (~)$ before it is fed into $K_{i} (~)$. The above operations are expressed in the following formulas:

1 $$\begin{aligned} y_{i}=\left\{ \begin{array}{lc} x_{i} &{} i=1; \\ K_{i}\left( x_{i}\right) &{} i=2; \\ K_{i}\left( x_{i}+y_{i-1}\right) &{} 2<i \le S. \end{array}\right. \end{aligned}$$

where $y_{i}$ denotes the output from each group, *S* denotes the configurable scaling dimension, and $K_{i} (~)$ denotes convolution. The outputs from the *S* groups are $y_{i}$ concatenated along the channel dimension and supplied to the CBAM attention mechanism. The feature maps are further improved using residual connections after they undergo a 1Ã-1 convolution to change the channel dimension. To replace the Bottleneck block, the Res2Attention block is finally incorporated into the CSPLayer.

### Loss function

The YOLOv5 loss function consists of three key elements: the target confidence loss, the classification loss, and the coordinate loss. The coordinate loss is computed using CIoU loss, whereas the target confidence loss, classification loss, and loss with logits are computed using BCE with logits loss.

By assigning different weights to feature maps of various scales, the weighted target confidence loss is used to make the model concentrate more on small objects during the detection phase. The output feature map sizes of 4.0, 1.0, and 0.4 correspond to 80Ã-80, 40Ã-40, and 20Ã-20, respectively. This highlights the necessity of high-resolution feature maps for increasing the precision of small item detection. Detected objects are accurately sorted into different groups by using the classification loss to evaluate the accuracy of the classification predictions. The positional variations between predicted boxes and ground-truth boxes are quantified using the coordinate loss. Given the nature of object detection tasks, it is crucial to consider target confidence, classification, and position information. The following shows the computation of the overall loss function:

2 $$\begin{aligned}  Loss=\lambda _{1}L_{obj} +\lambda _{2}L_{cls}+\lambda _{3}L_{loc} \end{aligned}$$

where the balancing coefficients, represented by the variables $\lambda _{1}$, $\lambda _{2}$, and $\lambda _{3}$, are used to adjust and balance the relative importance of different loss components in the overall loss function.

The following formula is used to determine the target confidence loss:

3 $$\begin{aligned} {{L}_{obj}}=-\sum \limits _{i=0}^{{{S}^{2}}}{\sum \limits _{j=0}^{B}{I_{ij}^{obj}\left[ {{\widehat{C}}_{i}}\log {{C}_{i}}+\left( 1-{{\widehat{C}}_{i}} \right) \log \left( 1-{{\widehat{C}}_{i}} \right) \right] }} -{{\lambda }_{noobj}}\sum \limits _{i=0}^{{{S}^{2}}}{\sum \limits _{j=0}^{B}{I_{ij}^{noobj}\left[ {{\widehat{C}}_{i}}\log {{C}_{i}}+\left( 1-{{\widehat{C}}_{i}} \right) \log \left( 1-{{\widehat{C}}_{i}} \right) \right] }} \end{aligned}$$

where $S^{2}$ denotes the number of separated grids, *B* denotes the number of anchor boxes in each grid, $I_{ij}^{noobj}$ denotes the presence of an object in the anticipated box, and it has a value of 0 or 1. The ground truth confidence score is represented by the letter $\widehat{C_{i}}$, and $C_{i}$ stands for the target’s projected confidence score. The penalty weight coefficient is represented by $\lambda _{noobj}$.

The following equation is used to determine the categorical loss:

4 $$\begin{aligned}  {{L}_{\text {cls}}}=-\sum \limits _{i=0}^{{{S}^{2}}}{I_{ij}^{obj}\sum \limits _{c\in classes}{\left\{ \widehat{{{P}_{i}}}\left( c \right) \log \left[ {{P}_{i}}\left( c \right) \right] +\left[ 1-\widehat{{{P}_{i}}}\left( c \right) \right] \log \left[ 1-{{P}_{i}}\left( c \right) \right] \right\} }} \end{aligned}$$

where $P_{i} (c)$ and $\widehat{P_{i}}(c)$ represent the probability values of the predicted and actual targets, respectively.

The CIoU Loss is used by YOLOv5 for bounding box regression. The coordinate loss in YOLOv5 is calculated using the following formula:

5 $$\begin{aligned} {{L}_{loc}}=1-IoU+\frac{{{\rho }^{2}}\left( b,{{b}_{gt}} \right) }{{{m}^{2}}}+\alpha \upsilon \end{aligned}$$

6 $$\begin{aligned} \alpha =\frac{\upsilon }{\left[ 1-IoU \right] +\upsilon } \end{aligned}$$

7 $$\begin{aligned} \upsilon =\frac{4}{{{\pi }^{2}}}{{\left( \arctan \frac{{{w}_{\text {gt}}}}{{{h}_{\text {gt}}}}-\arctan \frac{w}{h} \right) }^{2}} \end{aligned}$$

where *b* represents the centroid of the predicted frame, while $b_{gt}$ represents the centroid of the actual frame. The symbol $\rho$ denotes the Euclidean distance between these centroids. Besides, *m* represents the diagonal length of the bounding box that encloses both the predicted frame and the real frame, $\alpha$ denotes the weight coefficient, *IoU* represents the intersection over union, which calculates the ratio of the intersection area to the union area of the predicted and real frames’ bounding boxes. *v* represents the aspect ratio difference between the predicted box and the real box.

## Experiments and analysis

The proposed method was evaluated on three publicly available steel surface defect detection datasets that are widely used in the field of object detection: NEU-DET, GC10-DET and APDDD datasets. Through extensive experiments, this paper demonstrates the soundness, effectiveness, and superiority of the experimental design for the improved YOLOv5s.

### Experimental details

In this study, the experiment was carried out using the PyTorch deep learning framework, and the environment was set up on a Windows 10 operating system. The experiment’s hardware and software setup are as follows: Intel Core i5-12400F CPU, NVIDIA GeForce RTX 3060Ti G6X GPU, PyTorch 1.12.1, and CUDA 11.6. YOLOv5s was taken as the experiment’s preferred model. The SGD optimizer was used throughout the training process, with 500 epochs, an initial learning rate of 0.01, a momentum of 0.937, a weight decay factor of 0.005, a batch size of 8, and 1 worker for data loading.

### Datasets for evaluation

#### NEU-DET dataset

**Figure 7:**
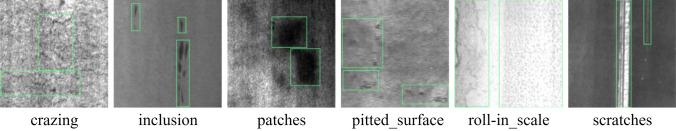
Annotated example images from the NEU-DET dataset show six different types of defects that have been found in steel strips.

The NEU-DET dataset^54^ is a collection of steel surface defect data gathered by Northeastern University and is mainly used for research on surface defect spotting and detection in hot-rolled steel strips. It includes 1800 grayscale images with a size of 200Ã-200 pixels. There are six common surface flaws in hot-rolled steel strips: crazing, inclusion, patches, pitted surface, rolled-in scale, and scratches. As illustrated in Fig. 7, the dataset includes 300 images for each category of defect, some of which may have several different types of faults.

#### GC10-DET dataset

**Figure 8:**
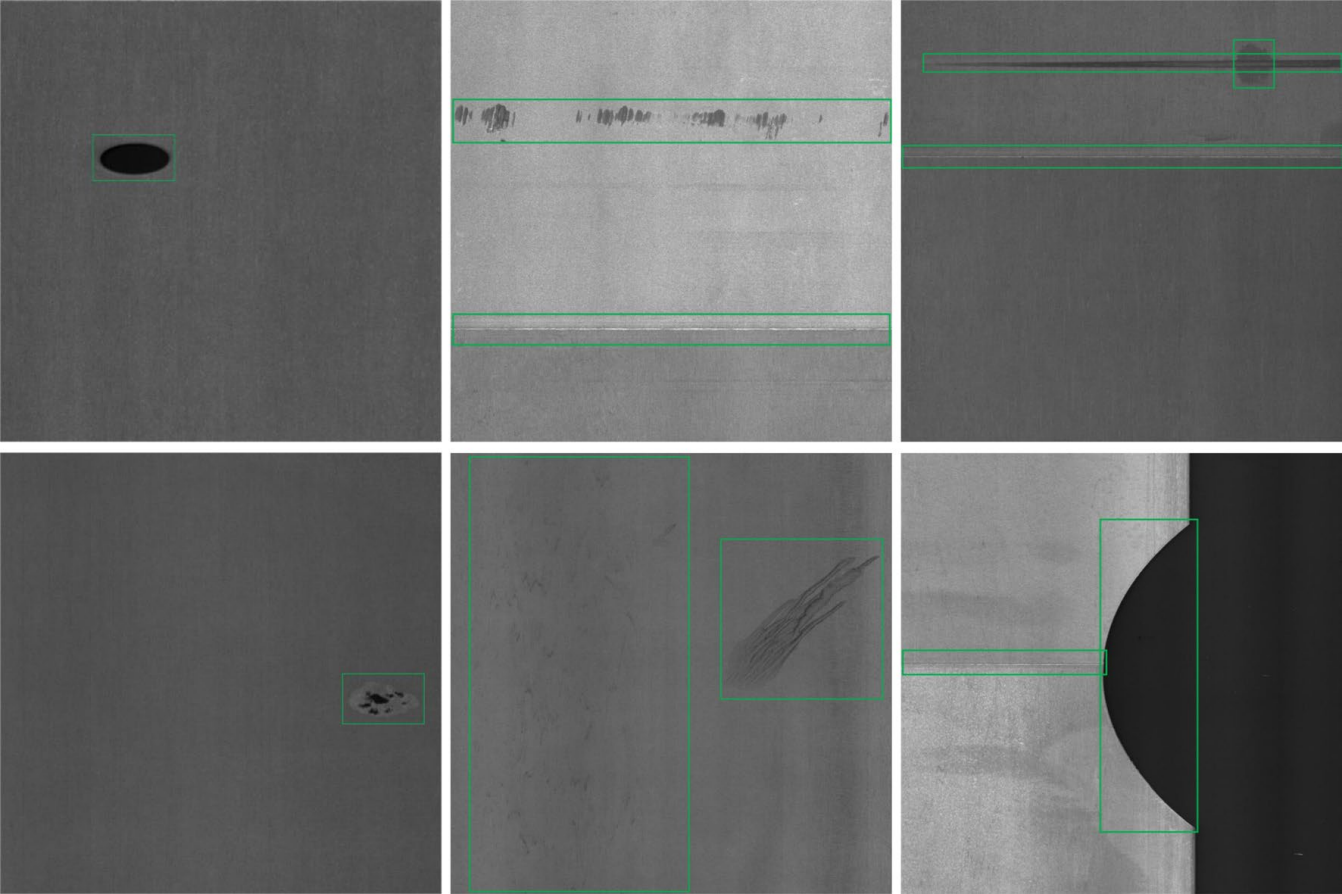
Labeled images of strip defects from the GC10-DET dataset.

The GC10-DET dataset^55^ was recently generated under actual industrial settings for extensive metal surface defect identification. It includes a total of 2300 images with a resolution of 2048Ã-1000 pixels. The dataset includes ten types of defects found on the surface of steel plates, including various punching hole, weld line, crescent gap, water spot, oil spot, silk spot, inclusion, rolled pit, crease, and waist folding. Figure 8 displays some defect sample images with annotations. With strong inter-class similarity and unbalanced sample distribution, the GC10-DET dataset shows a substantial variance in the number of images for each type of defect. Also, there could be multiple defect types in the same image, posing a challenge to defect detection algorithms due to the unbalanced data distribution.

#### Aluminum profile surface detection database

The image of the data set produced by the initial open source dataset of the 2018 Guangdong Industrial Intelligent Manufacturing Big Data Innovation Competition-Intelligent Algorithm competition “Aluminum Profile Surface Defect Identification”. APDDD is the name of the detection dataset. The APDDD dataset contains 1,885 defect samples, with a picture size of 2560 x 1920, and a total of ten different defect types, including dents, non-conductivity, scratches, orange peel, bottom leaks, bumps, pits, bumps, coating cracks, dirty spots.

Datasets are frequently stored in file formats including XML, TXT, CSV, etc. After downloading via the data availability download link, the NEU-DET and GC10-DET datasets utilized in the paper are automatically in XML file format, whereas the YOLOv5 model because the TXT file format is utilized, which is relatively easy, we must transform the file format in order to fulfill the requirements of testing and training the model. The approach used in this study is to divide the dataset into two groups: a training set and a test set. Refer to the following papers^56–58^, the network model is trained on about 70% of the data that are randomly selected, and the accuracy and robustness of the model are tested on the remaining 30% of the data, as shown in Table 1. Many of the defects in the datasets have relatively modest sizes and diverse irregular shapes and patterns. Meanwhile, the complex geometries of these defects and their high degree of similarity across defect categories require algorithms with higher resolution and sensitivity to detect and categorize these defects correctly. Determining the presence of steel surface defects is complicated due to the high complexity of the defects’ shapes and their tiny size. The developed algorithm must distinguish between tiny distinctions between defects and other textures or impurities on the steel surface as well as perform accurate defect recognition for various defect types. Additionally, the system must recognize and classify numerous defect types at once because some images may contain multiple types of defects.

**Table 1:** Details of the segmentation of the experimental datasets.

Dataset	Train set	Test set	Sum
NEU-DET	1260	540	1800
GC10-DET	1608	690	2298

### Metrics for evaluation

To evaluate the effectiveness of the improved model, this study uses four commonly used object detection model assessment measures: AP, mAP, GFLOPs and FPS. In AP, the precision of the model is evaluated for a given detection category by measuring the area under the precision-recall curve. mAP is the averaged AP score across all categories, and it provides a measurement of the overall detection accuracy. The quantity of floating point operations, or FLOPs for short, is a measure of computing level. It may be applied to quantify a model’s complexity. The frame-per-second (FPS) provides an assessment of the object detection model’s real-time performance by indicating the number of photos it can analyze in a second. The following provides the calculation formulas for recall and precision:

8 $$\begin{aligned}  Precision=\frac{TP}{TP+FP} \end{aligned}$$

9 $$\begin{aligned} Recall=\frac{TP}{TP+FN} \end{aligned}$$

10 $$\begin{aligned} AP=\frac{Precision+Recall}{2} \end{aligned}$$

11 $$\begin{aligned}  mAP=\frac{\sum \limits _{i=1}^{k}{A{{P}_{i}}}}{k} \end{aligned}$$

12 $$\begin{aligned} FPS=\frac{1}{\frac{1}{n} {\textstyle \sum _{i=1}^{n}t}_{j}} \end{aligned}$$

where the key measurements of the confusion matrix are TP, FP, and FN. TP denotes the number of true positive samples the classifier properly identified, FP denotes the number of false positive samples that the classifier reported, and FN denotes the number of false negative samples. The above formulas show that a higher mAP indicates better overall model performance. Here, *i* represents a particular class, while *k* represents the overall number of detection classes. The parameters *n* and *t* represent the number of pictures to be identified and the time taken to detect one image, respectively.

### Performance assessment

#### Comparison with state-of-the-art

In this research, the efficiency of the proposed model is evaluated using the NEU-DET dataset and the GC10-DET dataset. Existing one-stage and two-stage detection models are compared in terms of mAP. The NEU-DET dataset is taken as the basis for the initial comparison trials, and the comparison results of each model’s mAP and numerous default categories are listed in Table 2. Since small things like crazing make up most of the NEU-DET collection, it is essential to increase small object identification accuracy. The comparison between common one-stage approaches including SSD, RetinaNet^59^, FCOS^60^, and YOLO series (including YOLOv3, YOLOv3-spp, YOLOv4, and YOLOv8s) and two-stage approaches including Faster R-CNN, Cascade R-CNN^61^, and DDN^20^ show that the Faster R-CNN with VGG16 and ResNet50 as the backbone networks tend to achieve higher detection accuracy. In terms of performance against other fault categories, the improved model performs the best. The mAP of the improved model reaches 51.2%, 83.1%, 91.0%, 82.2%, 71.5%, and 91.8% for crazing, inclusion, patches, pitted surface, rolled-in scale, and scratches, respectively. Faster R-CNN with ResNet50^50^ as the backbone network marginally outperforms other approaches in crazing and rolled-in scale, while RrtinaNet outperforms other approaches in pitted surface, and YOLOv3-spp takes the lead in scratches. However, the results demonstrate that the improved YOLOv5s achieves an mAP of 78.5%, with 4.9% higher mAP than the baseline, showing the best overall average detection precision. Then, comparison experiments were conducted on the GC10-DET dataset for current advanced network models, such as Faster R-CNN, SSD, RetinaNet, YOLOv3, YOLOv3-spp, YOLOv4, and YOLOv8s, to further confirm the improved model’s robustness and generalization ability. The comprehensive comparison results of defect accuracy and mAP for each model are shown in Table 3. Compared to the baseline, the improved YOLOv5s achieves an average detection precision of 67.3% in terms of mAP. To sum up, the improved YOLOv5s model can accurately and promptly identify various steel strip defects in a variety of application settings, satisfying the practical needs of industrial defect detection. By contrasting the two parameters, GFLOPs and FPS, with those of other models using the NEU-DET and GC10-DET datasets, we were able to increase the model’s detection accuracy. The GFLOPs have gone up from Baseline, although they are still lower than in other models. The model’s frame rate per second has dropped. For more information, see Tables 2 and 3.

**Table 2:** Comparison results of different models on NEU-DET.

Method	Network	mAP (%)	AP (%)						GFLOPs	FPS
			Cr	In	Pa	Ps	Rs	Sc		
Baseline	CSPDarknet53	73.6	42.4	82.0	90.0	77.8	59.4	89.9	15.8	112
YOLOv3	Darknet53	72.8	45.6	77.8	85.4	79.4	58.1	90.3	154.6	43
YOLOV3-spp	Darknet53	72.5	41.1	79.7	90.6	70.8	61.1	92.1	155.5	36
YOLOv4	CSPDarknet53	70.8	41.6	72.7	83.1	76.4	59.6	91.4	29.9	60
YOLOv8s	CSPDarknet53	75.4	44.7	81.2	88.5	80.1	66.4	91.4	28.4	91
SSD	VGG16	74.8	46.9	75.9	90.6	83.8	67.3	84.1	30.7	32
Faster R-CNN	VGG16	72.3	42.9	67.9	84.9	79.1	68.8	89.9	200.9	18
Faster R-CNN	ResNet50	77.9	52.5	76.5	89.0	84.7	74.4	90.3	91.3	14
DDN	ResNet34	74.8	48.0	75.9	87.4	78.3	68.4	90.8	–	17
FCOS	ResNet50	71.3	44.1	76.1	86.5	79.8	63.3	78.2	80.6	40
Cascade R-CNN	ResNet50-FPN	73.3	38.3	76.0	88.4	81.3	67.8	88.2	119.0	12
RetinaNet	ResNet50	71.2	41.6	77.5	90.4	83.9	61.8	72.1	83.2	23
Our work	CSPDarknet53	78.5	51.2	83.1	91.0	82.2	71.5	91.8	20.1	54

**Table 3:** Comparison results with different models on GC10-DET.

Method	Network	AP (%)											GFLOPs	FPS
		mAP (%)	Pu	Wl	Cg	Ws	Os	Ss	In	Rp	Cr	Wf		
Baseline	CSPDarknet53	65.2	96.5	93.6	96.2	77.5	62.8	59.1	23.3	33.5	40.2	69.1	15.8	71
SSD	VGG16	56.3	94.8	89.3	90.8	65.8	55.0	45.4	18.2	12.4	20.5	70.5	30.7	47
RetinaNet	ResNet50	59.9	92.4	88.4	94.5	74.1	54.5	54.4	28.7	15.5	21.4	75.1	41.6	18
Faster R-CNN	ResNet50	60.8	82.2	78.0	95.4	69.2	57.7	58.3	24.8	29.2	30.7	82.6	52.8	14
YOLOv3	Darknet53	58.3	96.7	73.0	95.2	77.1	56.7	45.9	25.8	20.2	14.4	77.6	154.7	61
YOLOV3-spp	Darknet53	60.6	96.5	82.5	96.8	75.5	57.4	48.4	26.4	22.0	20.6	79.8	283.9	55
YOLOv4	CSPDarknet53	61.2	90.4	89.8	93.9	62.6	59.4	48.3	23.6	17.7	37.6	88.2	30.0	56
YOLOv8s	CSPDarknet53	66.9	97.0	87.7	96.4	84.7	65.8	56.0	24.3	30.0	45.9	81.5	28.7	60
Ours	CSPDarknet53	67.3	97.1	94.3	96.0	76.2	62.9	56.1	25.2	33.1	50.7	81.9	20.4	34

#### Ablation experiment

Ablation experiments were conducted to evaluate the impacts of MSF and CRA block and investigate the performance of the YOLOv5s model on the NEU-DET and GC10-DET datasets. Table 4 displays the results of the ablation experiments. To explore the impact of MSF, it can be seen that various feature fusion approaches can affect the model’s concentration on feature information and its power to collect fine features and identify small objects. In this study, the model’s exploitation of minute information was enhanced by the use of three different fusion approaches. According to the experimental results shown in Table 5, using these three fusion approaches on the NEU-DET dataset can enhance the model’s functionality. Specifically, fusion approaches a and method b improved the mAP by 2.4% and 2.5% over baseline, respectively. Then, the two approaches were combined to develop the third fusion approach, MSF, which outperformed fusion approaches a and b, leading to a 3% increase in mAP over the baseline. According to the test results, the proposed fusion approach may collect global and local detailed information more effectively, enrich defect feature information, and improve the performance of the model.

**Table 4:** Results of ablation experiments on different datasets.

	Ablation setting		mAP (%)	
	MSF	CRA	NEU-DET	GC10-DET
	$\times$	$\times$	73.6	65.2
	$\times$	$\surd$	76.1	66.8
	$\surd$	$\times$	76.6	66.2
Baseline	$\surd$	$\surd$	78.5	67.3

**Table 5:** Performance of different fusion methods on NEU-DET.

Method	FLOPs	Parameters	mAP (%)	AP (%)					
				Cr	In	Pa	Ps	Rs	Sc
Baseline	15.8 G	6.70 M	73.6	42.4	82.0	90.0	77.8	59.4	89.9
Baseline+a	17.0 G	6.79 M	76.0	43.7	83.8	89.8	80.1	68.9	89.4
Baseline+b	22.1 G	9.98 M	76.1	44.7	83.3	92.2	83.5	62.2	90.8
Baseline+a+b	23.2 G	10.07 M	76.6	47.1	81.8	91.4	82.0	66.4	91.1

**Table 6:** Comparison of different attention mechanisms on NEU-DET.

Attention	FLOPs	Parameters	mAP (%)	AP (%)					
				Cr	In	Pa	Ps	Rs	Sc
ECA	20.0 G	8.29 M	76.4	47.8	85.0	91.5	78.7	64.5	90.6
SE	20.0 G	8.32 M	77.0	47.6	84.0	91.4	78.1	69.8	91.0
EFSE	20.5 G	8.61 M	77.5	48.2	83.6	92.7	82.2	65.4	92.6
CBAM	20.1 G	8.33 M	78.5	51.2	83.1	91.0	82.2	71.5	91.8

Meanwhile, the research investigates the effects of various attention processes on the CRA block, building on the baseline that makes use of the MSF. ECA^62^, SE^63^, EffectiveSE^64^, and CBAM^65^ were tested on the NEU-DET dataset along with other attention mechanisms. The effect of using the attention mechanism is listed in Table 6. Note that each of these approaches helped to reduce the computational complexity and parameter quantity of the model. Particularly, compared to the baseline with MSF, the baseline with CBAM achieved 1.9% higher mAP. The examination of the experimental data indicates that the CBAM significantly improves the feature extraction capabilities of the model’s backbone when building the CRA block. It emphasizes capturing more features and mitigating the effect of noise, thereby greatly enhancing the model’s detecting capacity.

### Results of defect detection visualization

**Figure 9:**
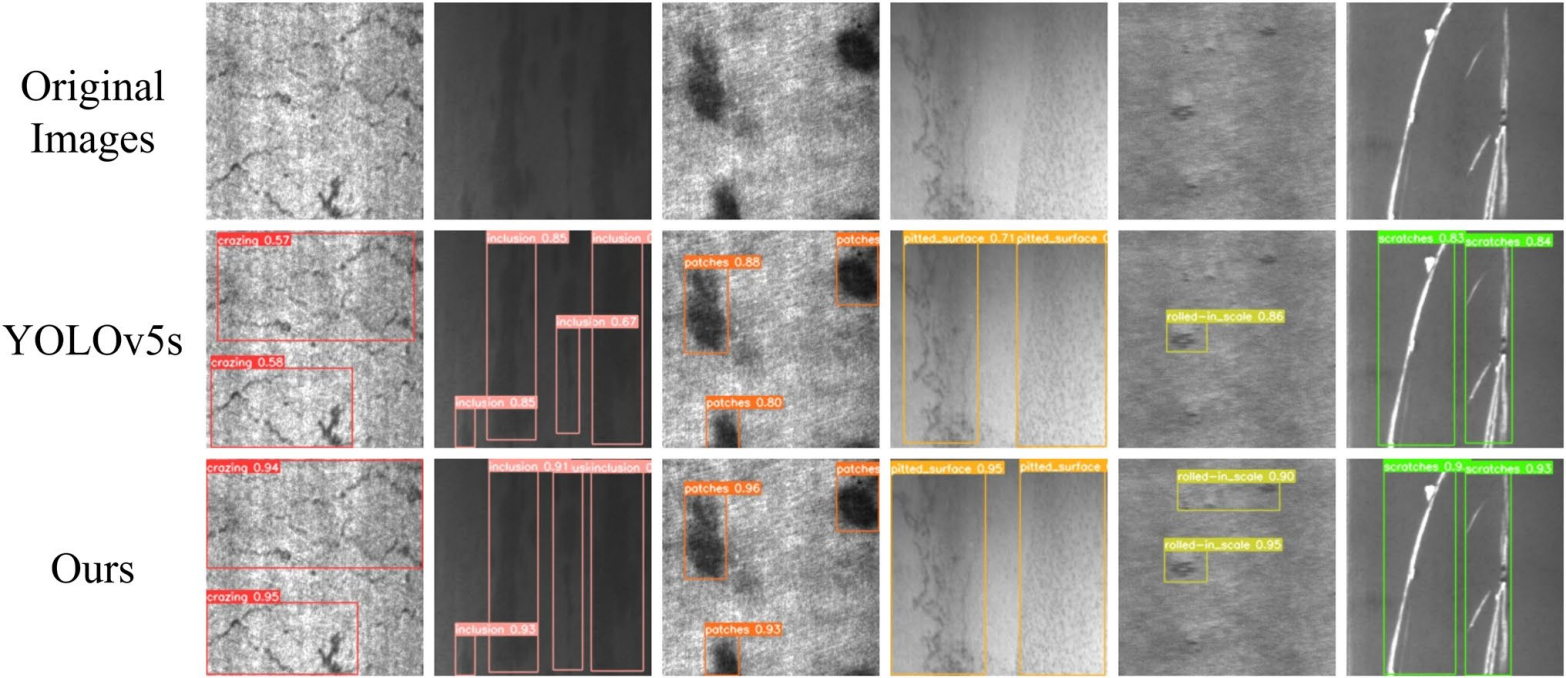
Visualization of the prediction results on the NEU-DET dataset.

**Figure 10:**
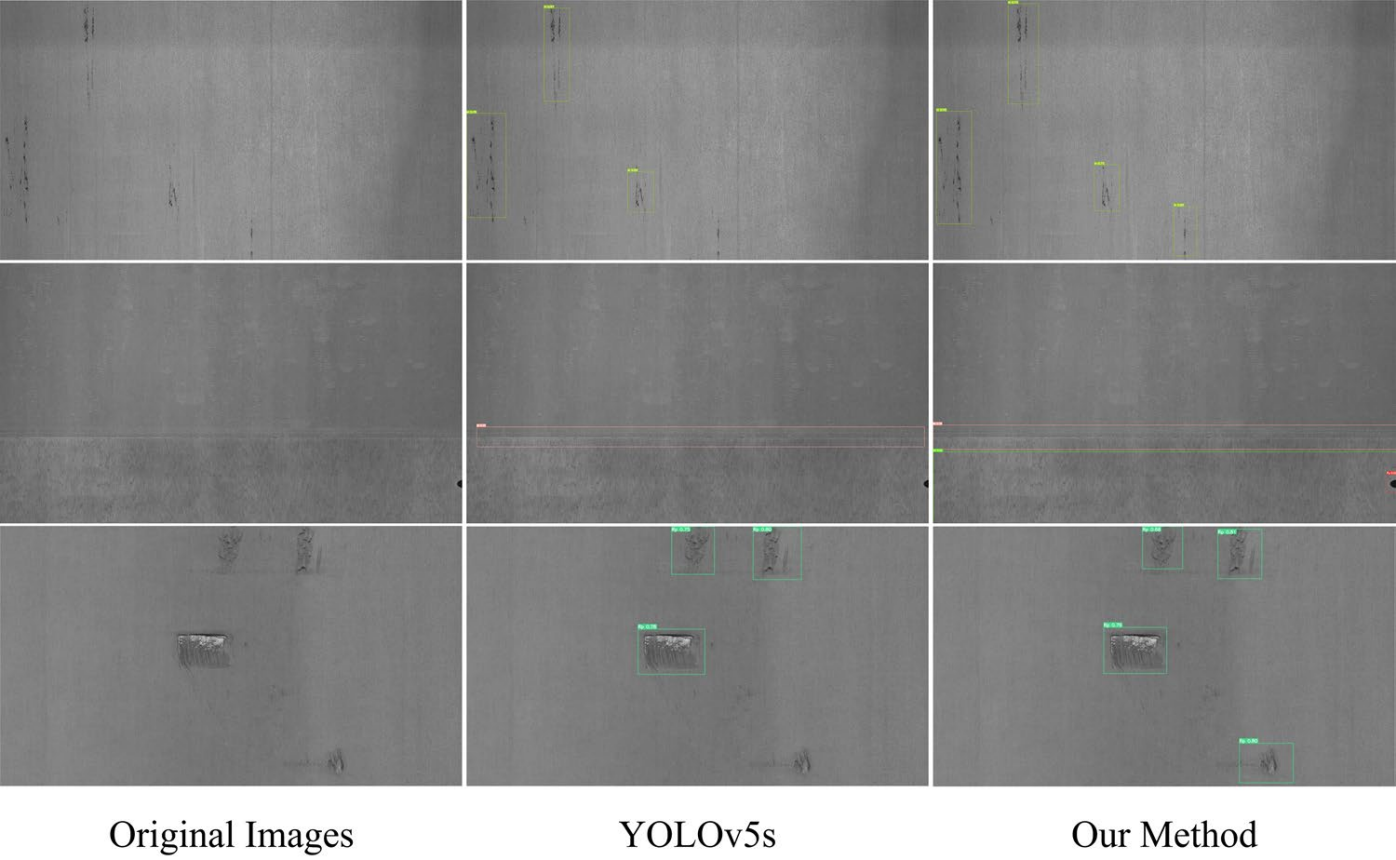
Visualization of the prediction results on the GC10-DET dataset.

A visual analysis was performed to investigate the model’s efficacy by comparing it with the baseline on the NEU-DET and GC10-DET datasets, and the results demonstrate the model’s performance intuitively. Figure 9 shows the predictions on the NEU-DET dataset, whereas Fig. 10 displays the predictions on the GC10-DET dataset. These plots provide a visual representation of how the model recognizes several types of defects in the images. These prediction results demonstrate that the proposed method achieves higher prediction accuracy and more exact localization of the defects than the baseline.

## Discussion

**Figure 11:**
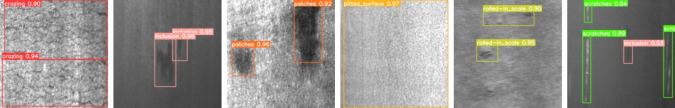
Analysis of different defects from NEU-DET.

During the industrial manufacture of strip steel, minor flaws including Cr, Pa, In, and Sc defects are frequently observed in the NEU-DET dataset, as shown in the Fig. 11. It is natural to notice from the images that many fault categories have tiny overall flaws. There are several and minor flaws. As the image illustrates, it is evident that addressing the issue of numerous minor faults is more important than addressing the large-area problems of Ps and Rs. Table 2 demonstrates how our technique increases the accuracy of tiny defect identification, and Fig. 9 illustrates how our method performs better and increases defect detection accuracy. Comparative tests were carried out on the APDDD dataset, as indicated in the Table 8, to confirm the scalability of our approach for defect identification in various materials. The findings in the table show that our approach came in second best highest ranking in mAP, suggesting that there may be room for growth with this approach. Furthermore, on two datasets, we compared the mAP and FPS parameters with those of other models. Table 7 shows that our approach produces the best results in terms of both mAP and FPS when compared to other approaches.

**Table 7:** Comparison results with different methods.

Dataset	Method	Backbone	mAP (%)	FPS
NEU-DET	Li et al.^58^	CSPDarknet53	74.7	–
	GA-RetinaNet^56^	ResNet50	72.8	8.6
	ACA-Net^56^	ResNet50	74.6	7.6
	GA-Faster R-CNN^56^	ResNet50	75.6	7.5
	ACA-Faster R-CNN^56^	ResNet50	76.4	6.0
	Our work	CSPDarknet53	78.5	54.0
GC10-DET	M-YOLOv3^57^	Darknet53	41.1	-
	GA-RetinaNet^56^	ResNet50	58.9	6.1
	ACA-Net^56^	ResNet50	60.5	5.9
	Our work	CSPDarknet53	67.3	34.0

**Table 8:** Comparative outcomes on APDDD dataset using various models.

Method	Backbone	mAP (%)	AP(%)									
			De	Nc	Sc	Op	Bl	Bp	Pi	Cp	Cc	Ds
RetinaNet	ResNet50	33.5	21.8	57.1	16.2	62.2	32.4	1.7	48.4	30.0	63.8	1.8
Faster R-CNN	VGG16	38.6	30.8	53.4	19.3	63.0	30.6	13.0	42.7	38.4	87.9	6.8
Faster R-CNN	ResNet50	41.4	50.8	50.4	21.0	67.4	25.8	12.8	36.5	84.6	59.7	4.8
YOLOv3-tiny	Darknet19	55.6	68.8	69.1	21.7	79.3	53.5	42.4	45.6	57.1	89.3	29.4
YOLOv4	CSPDarknet53	53.8	45.6	70.0	33.3	66.4	42.8	27.2	63.5	96.4	66.2	26.2
YOLOv7	E-ELAN	54.5	64.1	72.1	32.5	83.4	53.4	31.2	35.3	59.4	94.3	19.3
YOLOv8s	CSPDarknet53	63.5	68.0	76.4	43.0	83.0	58.0	65.6	59.9	61.2	91.9	28.5
Our work	CSPDarknet53	59.2	64.1	74.9	35.1	77.8	60.2	38.3	68.6	58.0	97.5	17.4

### Limitation and future work

Even though our strategy has produced excellent outcomes, there are still certain problems that require attention. Environmental variables might affect our model’s performance because not all situations may have been included in the datasets used for training and assessment. The effects of light, smoke, loud noises, and other environmental factors can be introduced. As an alternative, data augmentation of defect attributes, transfer learning, and pre-training on other data sets can be used to improve the model’s generalization ability. Future research will focus on the integration of real-time fault detection and field deployment models, which is essential for practical use in industrial hot-rolled steel manufacturing.

## Conclusion

This work proposes an improved lightweight one-stage detection model, YOLOv5s, which can make predictions with higher precision in response to the difficulties in the surface defect detection of steel strips and satisfy the practical application needs in industrial production. First, the MSF module is designed to enhance the model’s ability to recognize small objects and increase prediction accuracy at various sizes. This improves the feature representation capabilities of feature maps. Second, the CRA block, based on attention-focused aggregation, improves the backbone network’s feature extraction capability and makes the model pay more attention to fine-grained defect feature information, thereby further enhancing the model’s performance in detecting steel strip surface defects with high inter-class similarity and complex backgrounds. By reducing noise, it can improve defect localization and detection accuracy. Finally, extensive comparative experiments with advanced models were carried out on the steel strip surface defect detection datasets, NEU-DET, GC10-DET, and APDDD, to confirm the model’s performance advantage. The experimental results show that the proposed method performs better in detecting steel strip surface defects, achieving higher detection accuracy and better defect localization performance. The proposed model may be useful in industrial production because it can successfully address the issues of complicated backdrops and high inter-class similarity in defect identification.

## Data Availability

This study did not report any data. The proposed method was evaluated on three publicly available steel surface defect detection datasets that are widely used in the field of object detection: NEU-DET(http://faculty.neu.edu.cn/songkechen/zh_CN/zdylm/263270/list/), GC10-DET(https://www.kaggle.com/datasets/alex000kim/gc10det) and APDDD(https://tianchi.aliyun.com/dataset/148297).
